# Management and Reconstruction of a Helical Rim Defect With Exposed Cartilage

**Published:** 2013-04-26

**Authors:** Lily Daniali, Eric Payne, Matthew J. Trovato

**Affiliations:** ^a^New Jersey Medical School—University of Medicine and Dentistry of New Jersey, Newark, NJ; ^b^Medical City Children's Hospital; ^c^Dallas Plastic Surgery Institute, Dallas, Tex

**Figure:**
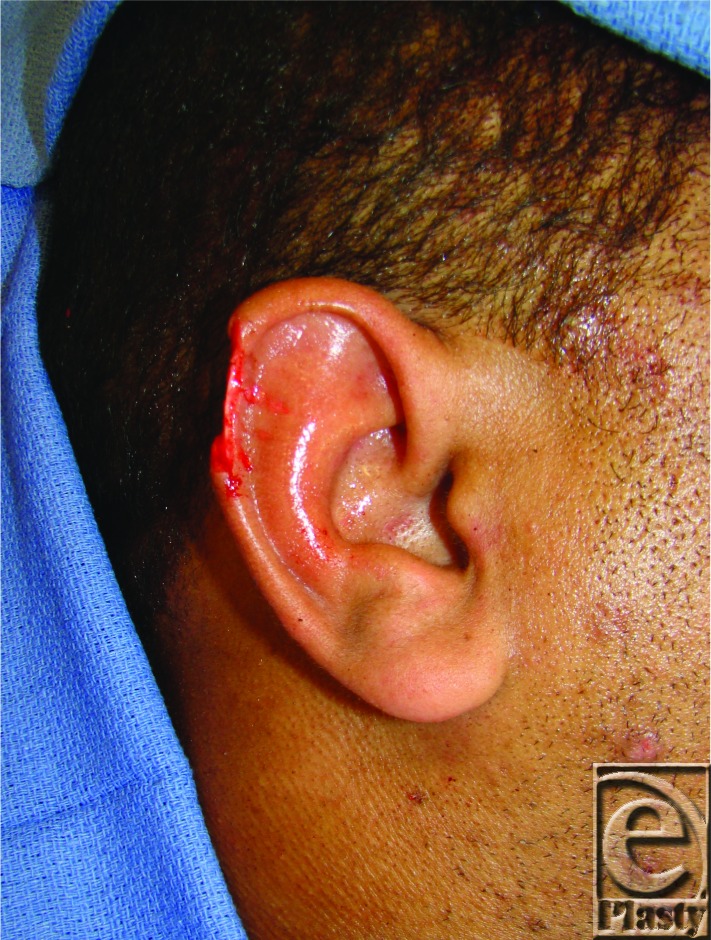


## DESCRIPTION

After sustaining a human bite to the right auricle, a 19-year-old man presented for management and reconstruction of a 2.6-cm isolated, full-thickness helical rim defect.

## QUESTIONS

1. **What is the appropriate management of this injury?**
2. **What complications may occur from a human bite to an ear?**
3. **What options are available for reconstruction of the upper half of the helix?**
4. **What is the minimal time until division of a delayed local flap pedicle?**

## DISCUSSION

Because of a higher concentration of oral flora, human bites carry the highest infection and complication rate of all mammalian bite injuries.^1^^-^3 Typically after a human bite, signs of local infection, such as cellulitis, localized pain, purulent drainage, or foul odor, may develop within 24 to 36 hours. Secondary to the excellent vascularity of the face, the occurrence of a facial bite infections is less likely than bites to other regions of the body. Human bites to the ear, particularly bites resulting in exposed cartilage, are an important exception to this general rule. Sixty-seven percent of human bites to the head and neck occur to the ear, and more than two-thirds of these patients present with exposed cartilage.^2^^,^^3^ After ensuring the patient has received adequate tetanus prophylaxis, copious high-pressure irrigation, and judicious wound debridement, the decision must be made to perform either primary (<24 hours after injury) or delayed closure of head and neck bite wound. While the literature is controversial, in general, there is an agreement that primary wound closure is the treatment of choice for all uninfected head and neck bites presenting within 24 hours from injury to maximize esthetic outcome. Nevertheless, human bites to the ear with exposed cartilage should be managed with extreme caution. A recent retrospective review of 44 human bites to the head and neck found that while 25% of human bites to the head and neck became infected, of these infections, 90% occurred in bite wounds with exposed cartilage. Also, there was a significant correlation between postoperative infection and primary closure. Finally, among patients' whose bite wounds were closed primarily, a failure to receive at least 48 hours of intravenous antibiotics was also associated with an increased risk of infection.^2^

Because of the higher infection rate of human bites, use of prophylactic antibiotic is accepted. On average, human bite wounds contain 5 different microorganisms.^3^ The most common infectious bacteria found in these polymicrobial wounds are *Staphylococcus aureus*, *Staphylococcus epidermidis*, viridans streptococci, *Eikenella corrodens*, *Haemophilus influenza*, and β-lactamase producing anaerobes such as *Bacteroides fragilis*. In adults, amoxicillin/clavulanate by mouth or clindamycin plus ciprofloxacin, in penicillin-allergic patients, is appropriate. The development of suppurative chondritis is the dreaded sequelae of ear soft tissue infections. It can result in deformation, resorption, and eventual destruction of the auricular cartilage framework. As a result, consideration should be given to admitting patients with human bites to the ear with exposed cartilage for 48 hours of intravenous antibiotic therapy.^2^^,^^3^ Patients should then be transitioned to an oral antibiotic regimen for a total 10-day course. Finally, transmission of viral diseases such as hepatitis B and C, herpes simplex, and human immunodeficiency virus through human bites has been reported.^4^

The patient's defect in this case is limited to the helix and lies on the border between the superior and central thirds of the helix. Several options exist for reconstruction of helical defects in this area. If the helical defect is less than 1.5 cm in width and extends from the rim into the scapha or triangular fossa, the defect can be converted into a wedge-shaped resection and can be closed primarily. The excision of small Burrow's triangles on the sides of the wedge resection from the antihelix aids in closure without the production of cupping.^5^ For defects between 1.5 and 2.5 cm in width, Antia-Buch helical chondrocutaneous advancement flaps allow for closure of the defect by creating further helical mobility.^6^ For isolated helical defects 2.5 cm and larger, the posterior auricular flap is a versatile, local workhorse flap. The posterior auricular flap can be transposed to reconstruct an isolated helical rim defects as either a superior or inferiorly based transposition flap or as an interpolated tube flap. In this particular case, the patient's defect was reconstructed with a superiorly based, transposition flap. In the first stage, the flap was raised and inset over the helical defect, but the pedicle was not divided during this operation. Although some authors believe this flap is an axial pattern flap, the majority of the literature considers its blood supply to be random. Three weeks later, once the transposed tissue developed adequate vascular ingrowth from the wound bed, the pedicle was divided. The donor site was closed primarily, and the flap was inset posteriorly to complete the helical contour.

The advantages of this reconstructive technique include the minimization of incisions on noninjured portions of the ear, utilization of retroauricular skin with an excellent color and texture match to auricular skin, and the ability to primarily close the hidden donor site. A recent case series analysis by Schonauer et al of 57 patients with posterior auricular flap reconstruction demonstrated uneventful healing in 95%. Two patients had minor flap tip necrosis that was managed conservatively, and 93% of patients felt they had an achieved a satisfactory esthetic result.^7^ With appropriate staging, the posterior auricular flap provides reliable, esthetic coverage of isolated helical rim defects.

## Figures and Tables

**Figure 1:**
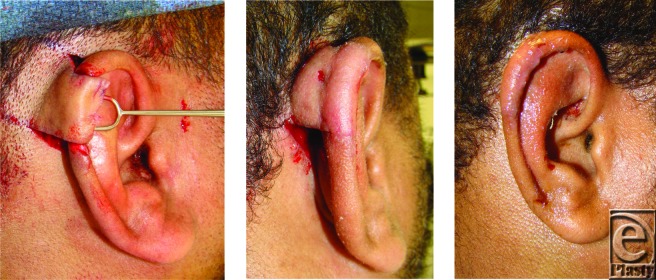
Intraoperative transposition of a superiorly based postauricular transposition flap (*left*). Three-week postoperative appearance prior to division of the pedicle (*center*). Final postoperative appearance (*right*).

## References

[1] Goldstein EJC (1992). Bite wounds and infection. Clin Infect Dis.

[2] Stierman KL, Lloyd KM, Luca-Pytell DM (2003). Treatment and outcome of human bites in the head and neck. Otolaryngol Head Neck Surg.

[3] Ambro BT, Wright RJ, Heffelfinger RN (2010). Management of bite wounds to the head and neck. Facial Plast Surg.

[4] Morgan M (2005). Hospital management of animal and human bites. J Hosp Infect.

[5] Baker SR, Baker SR (2007). Chapter 22: reconstruction of the auricle. Local Flaps in Facial Reconstruction.

[6] Thorne CH, Wilkes G (2012). Ear deformities, otoplasty, and ear reconstruction. Plast Reconstr Surg.

[7] Schonauer F, Vuppalapati G, Marlino S (2010). Versatility of the posterior auricular flap in partial ear reconstruction. Plast Reconstr Surg.

